# Biodegradable Orthopedic Magnesium-Calcium (MgCa) Alloys, Processing, and Corrosion Performance

**DOI:** 10.3390/ma5010135

**Published:** 2012-01-09

**Authors:** Meisam Salahshoor, Yuebin Guo

**Affiliations:** Department of Mechanical Engineering, The University of Alabama, Tuscaloosa, AL 35487, USA; E-Mail: msalahshoorpirsoltan@crimson.ua.edu

**Keywords:** magnesium, biomaterial, implant, manufacturing, surface integrity, corrosion

## Abstract

Magnesium-Calcium (Mg-Ca) alloy has received considerable attention as an emerging biodegradable implant material in orthopedic fixation applications. The biodegradable Mg-Ca alloys avoid stress shielding and secondary surgery inherent with permanent metallic implant materials. They also provide sufficient mechanical strength in load carrying applications as opposed to biopolymers. However, the key issue facing a biodegradable Mg-Ca implant is the fast corrosion in the human body environment. The ability to adjust degradation rate of Mg-Ca alloys is critical for the successful development of biodegradable orthopedic implants. This paper focuses on the functions and requirements of bone implants and critical issues of current implant biomaterials. Microstructures and mechanical properties of Mg-Ca alloys, and the unique properties of novel magnesium-calcium implant materials have been reviewed. Various manufacturing techniques to process Mg-Ca based alloys have been analyzed regarding their impacts on implant performance. Corrosion performance of Mg-Ca alloys processed by different manufacturing techniques was compared. In addition, the societal and economical impacts of developing biodegradable orthopedic implants have been emphasized.

## 1. Introduction

Several million people suffer bone fractures caused by accidents or diseases per year in the USA alone. In addition, the number of bone fractures caused by age-related diseases such as osteoporosis will rapidly escalate in the coming years due to the increase of life expectancy. Expenses exceeding $1.0 billion annually [1] had to be incurred in the national health system in 2004–2005. Many of these fractures are too complex for external medical treatment but have to be surgically fixed by internal bone implants such as the orthopedic implant. Current commercial permanent metallic implants, e.g., bone screws and plates, are made of titanium, stainless steel, and cobalt-chromium alloys. Current permanent metallic implants suffer two grand challenges, *i.e.*, “stress shielding” and “surgical interventions”. First, the permanent metallic implant materials are too stiff (Young’s modulus 100–200 GPa) compared to the adjacent cancellous bones (Young’s modulus 10–30 GPa). Internal loads will be mainly supported by the implants that shield tissues such as the bone from carrying the normal mechanical stresses. This “stress shielding” results in a number of critical clinical issues such as early implant loosening, damage to healing process and adjacent anatomical structures, skeleton thickening, and chronic inflammation. Second, metallic implants should be removed 1 or 2 years after the first surgery. Therefore, another surgical intervention is necessary with all the personal, medical, social, and economical consequences and costs. Biodegradable implants, which dissolve in the human organism, will be an ideal solution to the grand challenges of “stress shielding” and “surgical interventions”.

Previous *in vivo* studies [2,3,4,5,6,7] have shown that magnesium-calcium (Mg-Ca) alloys may be suitable as degradable biomaterial for use in medical implant. The close Young’s modulus between magnesium (40 GPa) and cancellous bones (Young’s modulus 10–30 GPa) has the potential to minimize stress shielding. Furthermore, magnesium, an essential element of the human organism, is biocompatible with the human body. However, the Achilles heel of a Mg-Ca implant is that it corrodes too fast in saline media such as in the environment of the human organism.

Answering these questions is critical for the development of the next generation of biodegradable implants, which has significant societal and economic impacts. While a number of methods such as heat treatment and alloying element have been tried to mitigate the corrosion rate of Mg alloys, the effects are very limited. The fabrication of a biodegradable Mg-based implant and corrosion control of the Mg-Ca implant through adjustable surface integrity are still in the infancy. This paper aims to review the works performed in last decade in processing Magnesium alloys especially Mg-Ca alloys and the achievements in controlling their corrosion performance.

## 2. Orthopedic Implants

**Figure 1:**
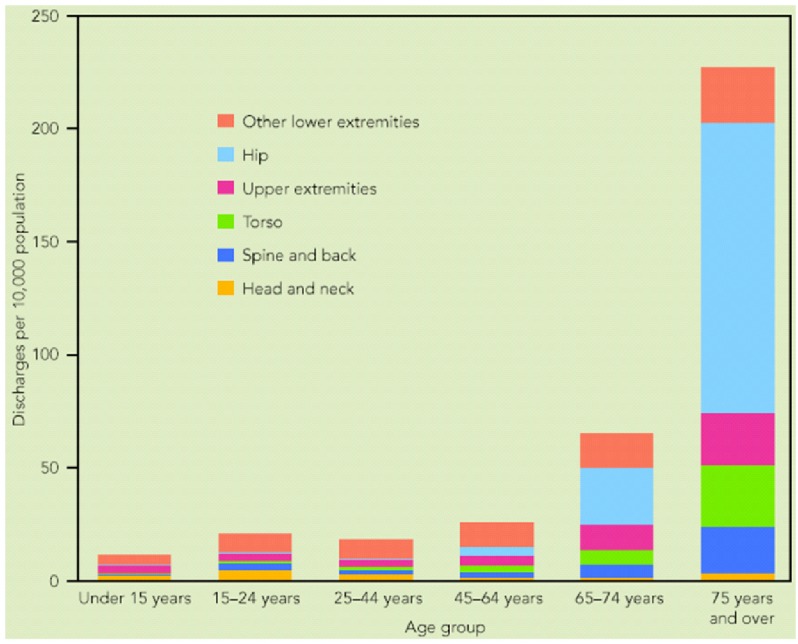
Hospital discharge rates for fractures, by age and body region of injury: USA, 2004–2005 [1].

Fractures in the skeletal system continue to be the leading cause of injury hospitalization in the United States, accounting for more than one-half of all injury hospitalizations in 2004–2005 [1]. For the older population, 75 years of age and over, almost three-quarters of injury hospitalizations were for fractures. As indicated in Figure 1, fractures in skeleton system can be categorized in four broad groups as fractures in head and neck, spine and back, torso, and extremities (upper and lower). It is noticeable that more than half of the fractures happen in extremities including shoulder, upper arm, forearm, elbow, wrist, hand, fingers, hip, upper leg, thigh, knees, lower leg, foot, ankle, and toes.

When a bone fractures, the fragments lose their alignment in the form of displacement or angulation. For the fractured bone to heal without any deformity the bony fragments must be re-aligned to their normal anatomical position. Orthopedic surgeons attempt to recreate the normal anatomy of the fractured bone by *reduction*.

In orthopedic surgery, implants may refer to devices that are placed over or within bones to hold a *fracture reduction* while prosthesis would be the more appropriate term for devices that replace a part or whole of a defunct joint. In this context, implants may be placed within or outside the body. Dental implants are one of the few medical devices which permanently cross the boundary between the inside and the outside of the body, since the base of the implant is connected to the bone of the lower or upper jaw and the top of the implant is in the mouth, where it can be crowned with an artificial tooth. 

## 3. Biodegradable Mg-Ca Orthopedic Biomaterials

**Figure 2:**
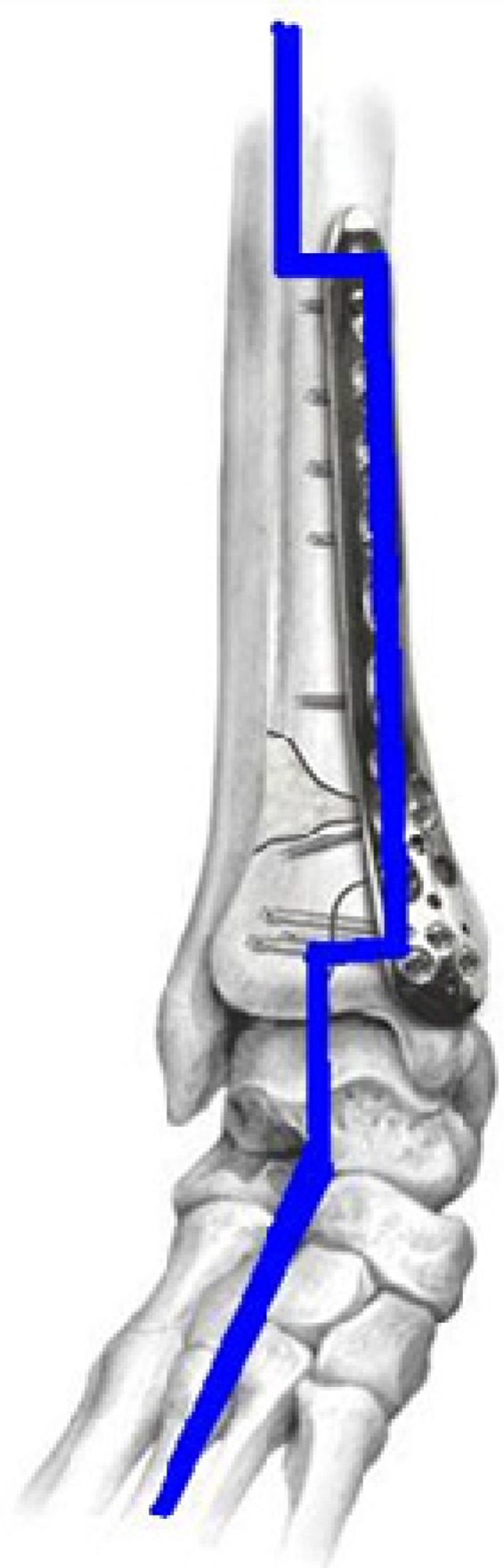
Stress shielding in locking compression plate fixing fractures in distal tibia.

Traditional methods of osteosynthesis or osteotomy use permanent metallic implants, e.g., bone screws and bone plates made of stainless steel, titanium, cobalt alloys [8,9,10,11,12]. The conventional metallic implants are too stiff compared to bones’ moduli. The modulus mismatch between permanent implants and bones shields the healing bone from being exposed to mechanical loads. This “stress shielding” (Figure 2) results in critical clinical issues such as early implant loosening, damage healing process and adjacent anatomical structures, skeleton thickening, and chronic inflammation [13,14,15,16,17,18,19,20,21,22]. Another issue for permanent implants is that revision surgeries are necessary when the bone heals. So personal, medical (the risk of refractures and additional days of after-treatment), social and economical consequences and costs have to be performed, in which the implants are excised. Only in patients older than 60 years it is acceptable to leave metal *in situ*. Usually, metal implants should be removed 1 to 2 years after the first operation [11,12]. In this context, Mg-based alloys, especially Mg-Ca alloys, have received a lot of attention due to their close modulus between Mg-based alloys and bones.

The first-generation biomaterials [23] were selected based on only two criteria: (i) suitable combination of physical properties to match those of the replaced tissue, and (ii) minimal toxic effect in human body [22]. The second generation of biomaterials encompasses two broad groups: (i) bioactive materials with the ability to interact with the biological environment in order to enhance the biological response as well as the tissue/surface bonding, and (ii) biodegradable materials with the ability to undergo a progressive degradation while new tissue regenerates and heals. Currently, biodegradable implants are mainly made of polymers, such as poly-L-Lactic acid. However, these polymer based implants usually does not have enough mechanical strength and consequently low load-bearing capacity which have led researchers to seek for biodegradable metallic substitutes. Mg and especially Mg-Ca alloys are among the most interested options.

Magnesium is an essential element to metabolic activities of the human body and intake of 300–400 mg of magnesium is normally required daily [24]. According to the measured weight loss rate (19–44 mg/cm^2^/day), as long as the total surface area of a magnesium implant is less than 9 cm^2^, the dissolved Mg^2+^would be easily absorbed or consumed by the human body [25]. However, the rapid generation of by-products such as hydrogen, hydroxide anion or OH^−^ could cause serious problems, even a fatal threat to a patient. Particularly, the severe side effect of rapid generation of hydrogen bubbles in the blood circulating system may finally exclude the possible application of magnesium stent in vascular system.

Alloying is one of the possible solutions to reduce the corrosion rate of Mg in the human body. A concern with alloying approach is biocompatibility of the alloying elements. Therefore unfavorable alloying elements largely limit the alloying approach and alloying elements should be carefully selected. Feser *et al.* [2] studied the influence of degradable Mg-Ca alloys with 0.6, 0.8, 1.0, and 1.2 wt % Ca on dendritic cell function. These cells are the major antigen representing the body cells. They concluded that Mg-Ca alloys have excellent biocompatibility and Mg^2+^ and Ca^2+^ cations produced as the result of *in vitro* degradation do not significantly interfere with dendritic cell functions. Li *et al.* [3] made binary Mg-Ca alloys with various Ca contents (1 to 3%) and different fabrication conditions for use as biodegradable materials within bone. The cytotoxicity evaluation using L-929 cells revealed that MgCa1.0 alloy did not induce toxicity to cells, and the viability of cells for MgCa1.0 alloy extraction medium was better than that for commercial pure c.p. Ti. They implanted MgCa1.0 alloy and c.p. Ti pins into the left and right rabbit femoral shafts and observed the degradation process for 1, 2, and 3 months. MgCa1.0 alloy pins had degraded gradually during the whole experiment period, which was evident by the reducing diameter of pins. At month 3, the MgCa1.0 alloy pin was totally absorbed and an irregular shaped hole was left in the implant position. Moreover, new bone was formed around the MgCa1.0 alloy pin while no remarkable radiographic signs indicating new bone formation was discovered around c.p. Ti pins during the experimental period. Gas shadows were observed around the MgCa1.0 alloy pins at month one, but they were vanished without any adverse effects in second month after operation. X-ray diffraction showed that the mineral phases in the precipitated white layer on MgCa1.0 pin were Mg(OH)_2_ and Hydroxy-Apatite (HA).

**Figure 3:**
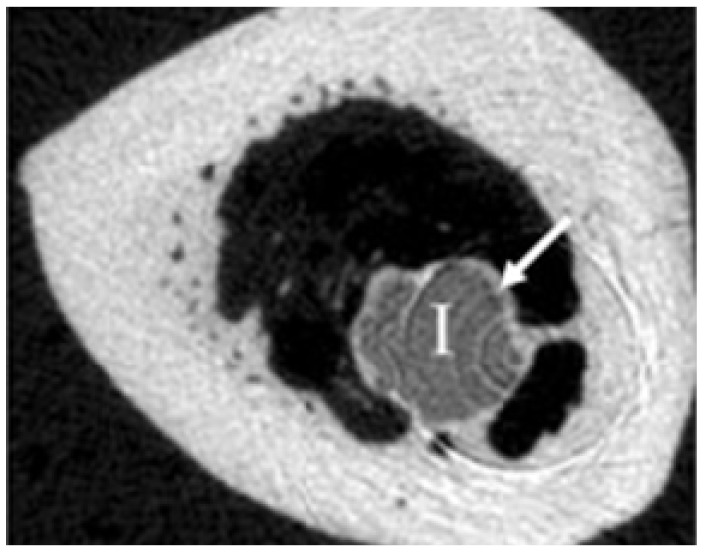
A ring of bone (arrow) has covered the MgCa0.8 implant surface 12 months after implantation [4].

Beside an adequate primary stability and the ability to degrade without side effects, a good biocompatibility is required to use Mg alloys as materials for osteosynthesis. Thomann *et al.* [4] conducted *in vivo* experiments to study effects of alloying Mg with calcium (in MgCa0.8), lithium, aluminum, and rare earth (in LAE442) elements on corrosion process. Extruded implants of these resorbable Mg alloys were implanted for a period of 12 month into the marrow cavity of both tibiae of New Zealand White rabbits. Figure 3 clearly shows a ring of bone covering the MgCa0.8 implant surface. This indicates the osteogenetic effect of this alloy. After 12 months the bone-implant contact was clearly stronger in MgCa0.8 case. However, it degraded more than LAE442 but slow enough to avoid any gas bubble generation. Stability of the implants is of major concern at beginning of the implantation. Thomann *et al.* [5] reported a pitting corrosion of MgCa0.8 implants after three months into implantation which increased till 6 months. MgCa0.8 implants showed an average loss in the cross section of more than half of the initial area after 12 months. The decrease in volume of MgCa0.8 implants were 11%, 31%, and 51% after 3, 6, and 12 months implantation, respectively.

## 4. Microstructure of Mg-Ca Alloys

**Figure 4:**
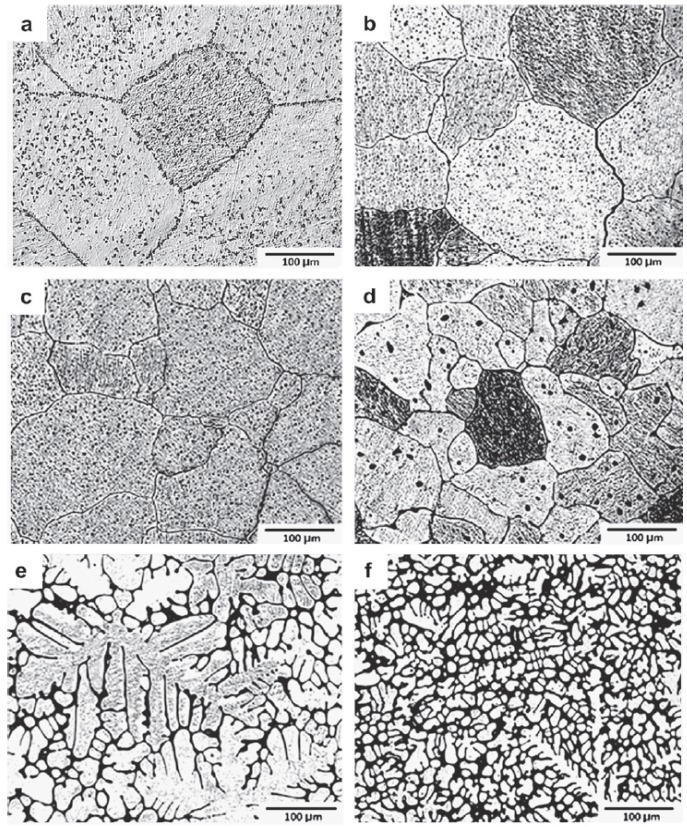
Optical micrographs of (**a**) pure Mg, (**b**) Mg-Ca0.5, (**c**) Mg-Ca1.25, (**d**) Mg-Ca2.5, (**e**) Mg-Ca5.0, and (**f**) Mg-Ca10.0 [30].

Binary alloy system of Mg-Ca has attracted a considerable attention in recent years to be used as implant material [3,11,12,26,27,28,29]. On the magnesium rich side of this system, the maximum solubility of calcium in the magnesium lattice at room temperature amounts to 0.8 wt %. At a calcium concentration of 16.2 wt % the alloy solidifies in eutectic composition. Low alloyed Mg-Ca systems consist of an α-phase solid solution (magnesium with interstitial calcium) and a eutectic structure (α phase + Mg_2_Ca). Electrochemically, Mg_2_Ca phase is more active than α-Mg and assumes the role of anode contradicting other intermetallics which are cathode in relation to Mg. Mg_2_Ca has the identical crystal structure as Mg, however, twice the lattice parameter magnitudes [29]. This reveals that Ca is a unique alloying addition to Mg in the context of biodegradable implants. The addition of a small amount of calcium has two distinct effects on Mg-Ca alloys. First, it increases the corrosion resistance and second, it minimizes the grain growth and leads to smaller grains in casts. A possible disadvantage of this grain refinement is the increased sensitivity to hot crack creation during the deformation process [27]. Rad *et al.* [30] studied the effect of calcium content on microstructure of Mg-Ca alloys containing 0.5, 1.25, 2.5, 5.0, and 10.0 wt % Ca (Figure 4). Their results revealed that the grain size and dendritic cell size decrease significantly with higher amounts of Ca while more Mg_2_Ca intermetallic phase appears in grain boundaries for higher Ca content. The mechanical behavior under dynamic and quasi-static conditions is influenced by the microstructure which in return is determined by the thermo-mechanical treatment history [31]. A fine grain structure possesses the lowest ductility and with increasing grain size ductility increases.

A high density of twins characterizes the micrograph in Mg alloys generally and in Mg-Ca alloys specifically. It seems that twining governs the onset of plastic deformation in Mg alloys. They are responsible for the higher compressive strain to failure in the quasi static compression test. However, the quasi static compression stress is lower than the dynamic compression stress. Knowing that twins’ density is lower in dynamic compression, this translates into the fact that twining increases the ductility of Mg alloys. The alloys in the as extruded condition have a very inhomogeneous distribution of grain size and after dynamic compression test the twin density in the coarse grains is higher. The competitive mechanisms of dislocation and twining lead to transcrystalline fracture in coarse grains and to crack propagation in fine grains. In non-extruded condition the grain size distribution is more homogeneous.

## 5. Mechanical Properties of Mg-Ca Alloys

**Figure 5:**
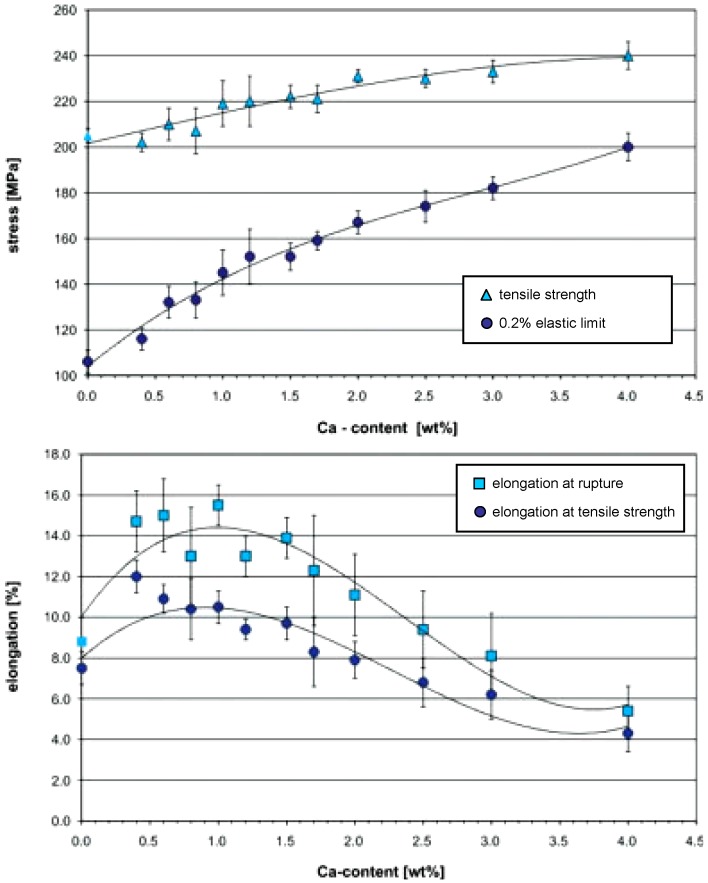
Tensile strength, 0.2% elastic limit, elongation at rupture, and elongation at the tensile strength of several Mg-Ca alloys [27,32].

Hassel *et al.* [27] and Drynda *et al.* [32] studied the influence of Ca amount on mechanical properties of Mg-Ca binary alloys. To produce this special Mg alloy, they used pure magnesium (99.8%) and Mg-Ca30.0 pre alloy (30.0 wt % Ca) and mixed them to produce Mg-Ca alloys with different calcium concentration. Alloying magnesium with low amounts of calcium, up to 4.0 wt %, leads to an increase in tensile strength up to approximately 240 MPa while the tensile strength of pure magnesium in extruded condition is about 200 MPa. The 0.2% elastic limit also increases steadily with increasing concentration of calcium. For low alloyed compositions, the 0.2% elastic limit is about 80 MPa lower than the tensile strength. This is an indication of relatively high plasticity (Figure 5). The difference between tensile strength and elastic limit decreases down to 40 MPa for higher concentrations of calcium. No significant increase in the tensile strength can be observed above 2.0 wt % calcium.

Moreover, the workability decreases and extrusion force increases significantly for higher amounts of calcium in direct extrusion. The processing of alloys containing more than 4.0 wt % calcium can only be done by indirect extrusion due to low workability and since the container friction, as a limiting factor, can be ignored in this case [27]. For higher amounts of calcium an increased amount of eutectic with a melting temperature of 516.5 °C can be observed. This may lead to considerable hot cracking if the deformation temperature is same as the melting temperature of the eutectic phase.

Investigations regarding the influence of Ca amount on plasticity and ductility of Mg-Ca alloys have shown that calcium content dramatically affects the elongation at rupture (Figure 5). For calcium concentrations below the solubility limit at room temperature, *i.e.*, 0.8 wt %, the elongation at rupture is about 13 to 15%. There is a continuous decrease in elongation above 1.5 wt % Ca. The alloy with the highest Ca content, *i.e.*, 4.0 wt %, has the lowest elongation of 5%. The elongation at the tensile strength decreases continuously from 12% at 0.4 wt % Ca to 4.5% at 4.0 wt % calcium. Precipitation of brittle Mg_2_Ca intermetallic phase on grain boundaries and inside the grains is responsible for poor ductility above 1.5 wt % Ca concentration. 

Forming and machining of brittle materials such as magnesium alloys with conventional methods are limited and intermediate treatments of the workpiece would be necessary. Under dynamic loading, metallic materials exhibit an increase in flow stress with increasing deformation rate. Materials of lower ductility can be formed up to large strains through implementing high strain rate deformation processes such as laser shock peening and high speed machining. Furthermore, constitutive material laws are necessary to describe the material behavior in simulation of high strain rate deformation processes [33,34,35,36,37,38]. The strain rate and temperature considerably influence the material flow behavior in deformation processes. Under quasi-static loading strain hardening causes an increase of force and acts as a stabilizing factor on deformation process. In case of dynamic loading, additional influences on the flow stress and the ductility of the material have to be taken into consideration. With increasing deformation rate, the strain rate sensitivity increases leading to a higher value of flow stress and stabilizes the deformation. On the other hand, the adiabatic character of the deformation process reduces the flow stress and promotes instability. Furthermore, the deformation process is influenced by inertia and mechanical wave propagation effects [39,40,41,42].

## 6. Ca Alloying and Surface Treatment Processes

Successful application of biodegradable Mg implants requires a controlled kinetics of degradation to avoid disruption of metabolic reactions during the healing process. As discussed in Section 4, alloying is one way to achieve that control on degradation. Further adjustment of degradation rate is mainly achieved through two methods: (i) surface coatings and (ii) mechanical treatments. Drynda *et al.* [32] developed binary fluoride-coated Mg-Ca alloys with improved degradation kinetics. The Ca content was 0.4, 0.6, 0.8, 1.2, and 2.0 wt % in their study. Electrochemical properties of uncoated and coated alloys were characterized in 0.5, 0.9 (physiological chloride concentration), 2.5, and 5.0% NaCl solutions at 37 °C. Solutions were buffered with tris-solution to a constant pH value of 7.4 (physiological pH value). Degradation rates were investigated using hydrogen evolution technique. Calcium concentration of 0.8 wt % resulted in the minimum degradation rate compared to other Ca contents. MgF_2_ coated Mg-Ca alloys showed slower rates (lower than 150 µA/cm^2^), regardless of the Ca content of the substrate Mg-Ca alloy. Moreover, no H_2_ gas formation was detected within the first 8 to 40 hours in fluorinated alloys. Thomann *et al.* [5] examined if the corrosion resistance of previously *in vivo* tested Mg-Ca0.8 implants could be increased by applying a magnesium fluoride coating. The gravimetric analysis before implantation, and 3 and 6 months after implantation showed a slight decrease in volume as compared to uncoated implants. The mean loss in volume after 3 months was 8.54% (±2.32%). After 6 months, this loss had approximately tripled (25.33% ± 12.66%). However, the mechanical properties of the coated implants exhibited a reduction in strength after 3 months. After 6 months, the strength of the coated implants was higher than that of uncoated cylinders.

Gu *et al.* [43] studied the effect of alkaline heat treatment on biocorrosion rate of Mg-Ca1.4 alloy. In this process coupons were soaked in three alkaline solutions (Na_2_HPO_4_, Na_2_CO_3_, and NaHCO_3_) for 24 h and then heat treated at 500 °C for 12 h. As the result, magnesium oxide layers with 13, 9, and 26 µm thick were formed on test surfaces, respectively, and *in vitro* corrosion of Mg-Ca1.4 alloy decreased effectively in simulated body fluid. This improvement in biocorrosion was in direct relation to oxide film thickness. The thicker film resulted in slower corrosion rate. Beside, the cytotoxicity with L-292 cells revealed no toxic effect of the formed oxide layers. Zhang *et al.* [44] coated Mg-Ca1.0 alloy with calcium phosphate using electrochemical deposition. The deposited calcium phosphate was mainly formed of flaky brushite crystallites. Electrochemical tests in Hank’s solution showed an increase in open circuit potential and a decrease in corrosion current density for coated samples indicating an enhancement in corrosion resistance. Moreover, coated samples produced much lower hydrogen initially but hydrogen evolution rate increased rapidly once pits happened in coatings. Li *et al.* [45] investigated the corrosion behavior of TiO_2_ coated Mg-Ca1.0 alloy in SBF. Uncoated Mg-Ca1.0 corroded considerably after 48 h immersion in SBF, however, the coated alloy remained almost intact after 168 h immersion except than a few break down sites. Corrosion current density of coated alloy was three orders of magnitude smaller than unprotected alloy. Although TiO_2_ is nontoxic and therefore biocompatible in that sense, however, in case of biodegradable Mg-Ca1.0 it is not clear if it could be absorbed or discharged by the body.

**Figure 6:**
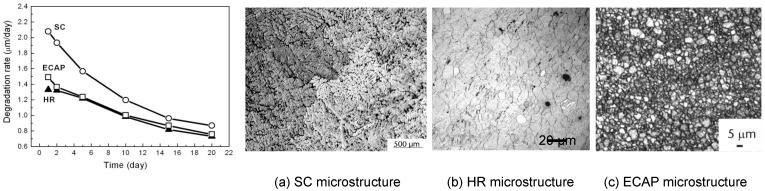
The effect of grain size on degradation rate of Mg alloy AZ31 [46]. (**a**) SC microstructure; (**b**) HR microstructure; (**c**) ECAP microstructure.

Another approach to enhance the bioperformance of Mg-Ca alloys is the mechanical treatment of the near surface or bulk material which will effectively reduce the corrosion rate. An advantage of using mechanical processing over surface coating is that it also enhances the material’s strength and fatigue resistance which are also critical in implant applications. The previous study [46] has shown that grain refinement may be a proper route to control the corrosion rate of Mg alloy AZ31 in Hank’s solution, see Figure 6. The samples were processed by squeeze casting (SC), hot rolling (HR), and equal channel angular pressing (ECAP), respectively. The corrosion rates of the HR and ECAP processed samples with fine grains (~10 µm) were only about 50% of the coarse grained (~400 µm) SC sample. However, fine grains alone would not increase corrosion rate. However, the effect of grain refinement for Mg-Ca alloys on corrosion has yet to be reported. 

Mechanical treatments like cutting and micro-forming are more focused on the near surface zone of the implants. Bach *et al.* [47] analyzed the influence of surface treatment, different alloy compositions and varying heat treatments on corrosion kinetics of work piece surfaces in salt spray corrosion tests according to DIN 50021 SS. Lower feed rates for a constant cutting speed resulted in harder layers close to surface caused by longer exposure to thermo-mechanical loads and therefore higher chances for microstructural changes. Higher cutting speeds for a constant feed rate gave rise to a smoother surface due to thermally induced reduction of the material strength on the shear plane and the increased easiness of cut. They ended up with more surface roughness using polycrystalline diamond (PCD) inserts than cemented carbides. The corrosion rate of the samples machined with PCD inserts was significantly higher than the corrosion rate of the samples machined with uncoated cemented carbide inserts. The more corrosion resistance was related to lower surface roughness produced by cemented carbide tools. It was also observed that increasing cutting speed intensifies the corrosion rate. However, the corrosion test with 5.0 wt % salt solution is too aggressive and only indicates the tendency.

Denkena *et al.* [12] sought to adjust the degradation kinetics of Mg-Ca3.0 alloy through modifying surface (*i.e.*, topography) and subsurface (*i.e.*, residual stresses) characteristics by applying turning process. They explored different combinations of cutting process parameters to create a specific degradation profile appropriate for certain medical application. They noticed that for same amount of depth of cut and feed, lower cutting speed shows lower corrosion rate and they related that to higher cutting forces and consequently higher and deeper compressive residual stresses. However, the better surface finish at lower feed rates did not show a significant influence on corrosion resistance.

Von Der Hoh *et al.* [6] examined the degradation behavior of Mg-Ca0.8 implants with different surface conditions for 3 to 6 months *in vivo*. The employed Mg-Ca0.8 implants received turning, sand-blasting, and threading treatments. Roughness depths were 3.65 µm for turned implants and 32.7 µm for sand-blasted implants. New bone formation and hydrogen gas evolution were used as metrics to study the degradation behavior. Their study could confirm the generally good clinical compatibility and osteoinductive potency of Mg-Ca0.8 alloy. Increase in *surface roughness* led to a faster degradation rate. However, a univaribale analysis of variance with significance level of 0.05 showed that the influence of the surface roughness was statically not significant (*p* = 0.257). The faster decomposition of Mg implants leads to more H_2_ gas release. The sand-blasted cylinders decomposed the fastest. Furthermore, 53% of the radiographically detectable gas generation appeared in sand-blasted implants. The turned cylinders which exhibited the least structural loss showed hardly any gas formation. The formed gas was resorbed in all cases except one without any clinical problems. Moreover, they found that Mg-Ca0.8 shows obvious pitting corrosion for threaded cylinders but not for turned and sand-blasted implants. In addition, turned implants showed the best integration into the bone compared to sand-blasted and threaded cylinders. Therefore, it seems a smooth surface is particularly suitable when using resorbable implants made of Mg alloys as bone implants in osteosynthesis*.* This is against conventional implants which will make better and stronger bone-implant bonding if the implant surface is rougher.

Literature survey on mechanical surface treatments reveals another process named deep rolling or shortly DR. This process, which is developed by Ecoroll, is very similar to the low plasticity burnishing (LPB) process developed by Lambda Technologies in terms of working principal [48], even though it is claimed that DR produces more cold work than LPB [49]. Using LPB/DR in medical device manufacturing applications is new and there are only a few published research works for that [50,51,52]. Denkena *et al.* [12] tried to control the corrosion of the Mg-Ca3.0 implant by mechanical treating the implant surface using DR technique and ultimately to achieve adaptable degradation profile for various medical applications. As compared to shot peening, LPB/DR avoids the contamination of the processed surfaces and consequently prevents the third body wear [53]. Besides, LPB/DR produces higher and deeper compressive residual stresses with less amount of clod work [49,54]. High levels of cold work leave a severely deformed surface layer with a high dislocation density that adversely affects the thermal and mechanical stability of compressive layer. However, Gill *et al.* [55] reported lower levels of cold work as a promising potential of DR where they compared this process with shot peening (SP) in terms of compressive residual stress and amount of cold work.

There is a general consensus [55,56,57,58,59,60,61] that more stable compressive residual stresses will be achieved by minimizing the amount of cold work necessary to generate them. Severe plastic deformation in shot peening makes compressive residual stresses less stable. LPB/DR could be an effective means of mitigating corrosion initiated failures without altering either material or design of the implants [62]. The depth of compression from LPB/DR can greatly exceed the maximum corrosion pit depth in orthopedic implants and therefore prevent failure from pitting or cracking and ensure safe-life operation of the implant [63]. LPB/DR is a novel process and it needs an extensive study of the effect(s) of various process parameters, e.g., lateral feed, speed, force, ball diameter, tool path, lubricant, *etc*., on surface integrity and product performance to develop the required database for different materials [64,65,66].

## 7. Corrosion Property of Mg-Ca Alloys

The basic electrochemical character of the magnesium with standard potential of −2.375 volts leads to a low corrosion resistance. Magnesium implants’ surface passivates and builds up a thin grey layer of magnesium oxide, when exposed to air, which prevents further chemical reactions. However, magnesium is attacked significantly in saline media such as human body environment. These characteristics primarily enable Mg alloys particularly Mg-Ca alloys to be used as absorbable implant materials [11]. Magnesium can be entirely absorbed in human body like absorbable polymers when it is considered to be used in biomedical applications. Meanwhile, it offers the great advantage of higher mechanical strength as opposed to biopolymers. Dissolution of magnesium in chloride containing media like human body happens through the following reaction [27]:

Mg + 2H_2_O → Mg (OH)_2_ + H_2_↑

**Figure 7:**
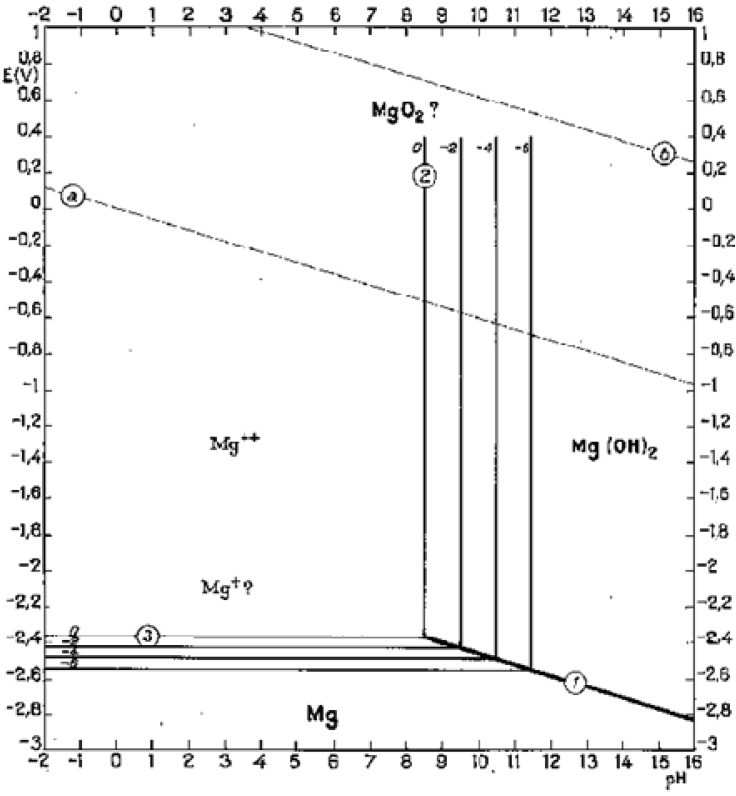
Potential-pH equilibrium diagram for magnesium-water system at 25 °C [67].

Magnesium reacts with water, which is plentiful in body fluid, and produces hydroxide and hydrogen. In high pH (>11.5) environments, magnesium hydroxide will play as a stable protective layer on the surface of magnesium implants, but lower pH (<11.5) will facilitate corrosion of magnesium alloys in aqueous solution (Figure 7) [67]. Since the local pH at implant-bone interface is about 7.4 or even lower due to secondary acidosis resulting from metabolic and resorptive processes after surgery [68], the magnesium hydroxide layer cannot cover the implant surface. Therefore, the constant exposure to high chloride containing electrolyte of the physiological system causes an accelerated corrosion on the Mg implant surface *in vivo*.

**Figure 8:**
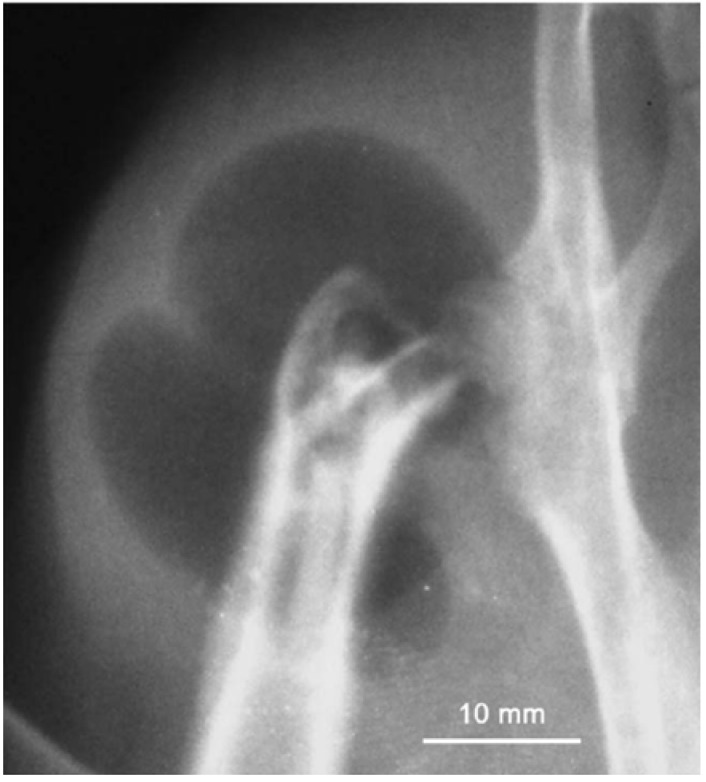
Subcutaneous gas bubble observed on postoperative radiographs for 4 weeks during magnesium implant degradation [68].

During magnesium dissolution, hydrogen gas is produced and actually hydrogen evolution rate equals to magnesium implant dissolution/corrosion rate. As such, eudiometry of hydrogen has been used as a tool to study long term degradation behavior of Mg-Ca implants *in vitro*. Corrosion of one gram magnesium results in production of 1.081 liter hydrogen gas [27,68]. The accelerated corrosion will lead to early loss of mechanical integrity and to generate hydrogen at rates too fast for human body to deal with which will result in the formation of subcutaneous gas bubbles [68,69]. Figure 8 shows a typical gas bubble containing hydrogen. As mentioned before, several possibilities exist to adjust the corrosion rate and two of them are using alloying elements and protective coatings. In alloying approach, critical issues are using minimized alloying elements, non-toxic elements, and biological compatibility. Magnesium alloys exhibit different degradation rates depending on the alloying element they contain. Most alloying elements such as aluminum and zinc are suggested to increase the rate of oxidation, while alloying magnesium with rare earth elements is suggested to decrease the oxidation rate of magnesium alloys [68].

Hassel *et al.* [27] studied the influence of Ca amount and chloride concentration in corroding media on corrosion behavior of Mg-Ca alloys. A negative shift of about −0.1 volts in open circuit potential (OCP) was observed by increasing chloride concentration in the electrolyte for all the investigated alloys. Polarization curves showed that with increasing amount of Ca in the alloy, mixed corrosion potential (E_corr_) becomes more noble or positive. The magnitude of the corrosion current density (i_corr_) also changed with Ca content and the higher the Ca content was, the larger the current density became. NaCl concentration showed similar effect and increased amount of chloride led to higher corrosion current density and corrosion rates. The eudiometric investigations revealed that all tested Mg-Ca alloys corrode slower than pure magnesium and hydrogen evolution rate or corrosion rate depends strongly on electrolyte concentration. In Mg-Ca2.0 alloy case, increase in NaCl concentration up to 0.9% led to increase in hydrogen formation. Between 0.9% to 2.5% chloride concentrations, the gas formation stayed almost constant and decreased by further increasing the chloride content up to 5%. The same trend was observed for other Ca amounts in Mg-Ca binary system. Decrease in the gas formation under high chloride contents was related to a layer of precipitated corrosion products which were rich in chloride. This layer covers the implant’s surface and weakens the chemical attack of the electrolyte. However, thick and white corrosion products precipitated on the specimen surface were not characterized.

Rad *et al.* [30] studied the effect of Ca content on *in vitro* corrosion behavior of Mg-Ca alloys with varying Ca content ranging from 0.5 to 10.0 wt %. Corrosion rate of Mg-Ca alloys increased significantly with higher amounts of Ca. This decrease in corrosion resistance was related to more Mg_2_Ca intermetallic phase formed in grain boundaries under high Ca contents. Higher corrosion rates led to higher pH value during the tests. Corrosion was more moderate and uniform in low Ca content alloys. Kirkland *et al.* [29] also investigated the influence of increasing calcium (Ca) content in binary Mg-Ca alloys along with the composition of the bio-fluid on *in vitro* degradation rate. Different Ca contents of 0.4, 0.8, 1.34, 5.0, 10.0, 16.2, and 28.0 wt % were considered. Three different bio-fluids were used starting with regular SBF and progressing to more human serum-like fluids by adding amino acids and vitamins (the second bio-fluid) and finally proteins (the third bio-fluid) to SBF. All three bio-fluids were buffered and the pH and temperature values were kept constant at 7.15 ± 0.05 and 37 °C ± 1 °C, respectively. The bio-fluid volume to exposed area ratio was 300 mL:1 cm^2^. It was observed that above solid solubility limit of ~1.34 wt % Ca, the corrosion rate increased with increasing Ca content and the corrosion potential became more negative. However, below the solubility limit, the corrosion rate stayed the same or even slightly decreased with increasing Ca additions. As the bio-fluid mimicked the human serum more closely, corrosion rate decreased between 10 to 290% and corrosion potential became more positive. This emphasizes that using more physiologically relevant test environments are necessary to effectively study the *in vitro* biodegradation behavior of Mg-Ca alloys.

Krause *et al.* [7] investigated the initial mechanical strength and *in vivo* degradation behavior of Mg-Ca0.8, LAE442, and WE42 alloys along with the change in their mechanical properties due to degradation. Mg-Ca0.8 showed an insufficient initial strength and a fast degradation. However, its ductility was constant during degradation and the degradation products were naturally occurring components in the human organism. LAE442 degraded slower and its initial strength seemed sufficient for load-bearing applications. However, rare earth alloying elements present in LAE442 resulted in products which their biocompatibility has to be studied yet. They did not recommend WE43 as an orthopedic material for fracture repair due to its heterogeneous and unpredictable degradation behavior.

Kannan and Raman [28] examined the degradation behavior and mechanical integrity of calcium-containing Mg alloys using electrochemical techniques and slow strain rate test (SSRT) method, respectively, in modified-SBF. AZ91Ca (1.0 wt % Ca), AZ61Ca (0.4 wt % Ca), and AZ91 (without Ca) were used. Potentiodynamic polarization tests were performed at 36.5 ± 0.5 °C in m-SBF buffered at 7.4. The general and pitting corrosion resistance of calcium-containing Mg alloys in m-SBF was significantly improved as compared to the base alloy. The corrosion current was significantly lower in AZ91Ca alloy than that in AZ91 alloy. Furthermore, AZ91Ca alloy exhibited a five-fold increase in the surface film resistance than AZ91 alloy. The mechanical properties of the Ca-containing Mg alloys decreased only by ~15% (for ultimate strength) and 20% (for elongation at fracture) in m-SBF as compared to these properties in air.

Rapid corrosion is almost an intrinsic response of magnesium to a chloride containing solution, like the human body fluid or plasma. Corrosion in bone plates mostly occurs at the plate-screw interface [70]. Previous experimental investigations have shown a correlation between cutting and non-cutting processes and the corrosion behavior of different magnesium alloys [47,71]. Theoretically, a bio degradable material should have a controllable dissolution rate or a delayed corrosion process. An implant made of such a material should be able to fully function before surgical region recovers or heals. After that, the implant should be gradually dissolved, consumed or absorbed. Obviously, a suitable corrosion rate is critical to a biodegradable Mg-Ca implant. To make this possible understanding the corrosion behavior of Mg-Ca alloys in body fluids is an essential step.

Bach *et al.* [47] used correlated noise measuring technique (CorrELNoise) in addition to gravimetric method to characterize the corrosion behavior of different surface conditions which were generated in milling under various process parameters. The aim was to find the correlation between different machining parameters and corrosion rate. The CorrELNoise has adequate sensitivity to analyze the initial corrosion events on the influenced test surfaces [72]. The sample which was machined with higher cutting speed (2,400 m/min) showed a stronger corrosion attack at the surface.

Denkena and Lucas [12] studied the possibility of adjusting degradation kinetics of biocompatible Mg-Ca3.0 implants through adjusting surface and subsurface properties by machining processes. They focused on differences in subsurface properties while similar surface properties were achieved. Turning and deep rolling processes were used to change subsurface properties especially residual stresses. The process parameters were selected in a way to achieve comparable surface properties especially surface roughness. The deep rolled surfaces were smoother than the turned surfaces. However, the turned surfaces were still located in a comparable range of finish quality. Significant differences occurred in subsurface residual stresses. Larger and deeper compressive residual stresses were achieved by deep rolling. Changes in microstructure (compression of grains) especially at maximum residual stress area were visible. However a significant modification in microhardness was not detected. The degradation behavior was determined through mass loss in 0.9 wt % NaCl solution (representing the salt content of body fluid). To determine the mass loss due to degradation, the hydrogen gas evolved as the result of Mg oxidation/dissolution was collected and the corresponding mass of degraded Mg was calculated using stoichiometry in the chemical reaction, *i.e.*, Mg + H_2_O → Mg(OH)_2_ + H_2_. The pH value of the solution was kept constant by a compensating reservoir and a periodic change of solution. The corrosion rates of the turned surfaces were *approximately 100 times* faster than the corrosion rates of the deep rolled surfaces. Within the range of surface qualities applied in their study, the better surface finish at low feed rate had no significant influence on the corrosion resistance. A homogeneous corrosion attack with moderate corrosion rates was observed in deep rolled surfaces. The corrosion resistance was improved until the modified layer of subsurface was dissolved and the unmodified bulk region was uncovered.

The corrosion mechanism is important, particularly with regard to the biomechanical properties of medical implant devices. Mg-alloys, in general, tend to pitting corrosion, especially close to chloride ions. In contrast, carbonate ions are able to suppress pitting corrosion totally. Calcium addition to Mg-based alloys enhances their general and pitting corrosion resistance significantly. Up to 0.8 wt % Ca, Mg-Ca alloys show a homogenous texture and a uniformly distributed corrosion. Higher percentages of calcium lead to irregular and more widespread corrosion [6].

The corrosion process depends not only on the element composition of the biomaterial and its processing, but also on the *corrosive environment* to which magnesium alloys are subjected. *In vivo* environment has two basic and important characteristics: it contains chloride anions (saline media includes NaCl) and its pH is 7.4. According to Pourbaix diagram (Figure 7), the passivating magnesium hydroxide (Mg(OH)_2_) cannot be stable and it will dissolve in body fluid producing Mg cations and hydroxide anions if pH stays at 7.4. Therefore, it seems an appropriate way to control Mg corrosion is to increase the surrounding pH in order to stabilize the products in the corrosion layer. Calcium has been known for years to reduce the susceptibility of magnesium to corrode when added in amounts of a few tenths of weight percents [73]. Gu *et al.* [74] studied the influence of artificial biological fluid composition on the biocorrosion of potential orthopedic Mg-Ca, AZ31, and AZ91 alloys. Their results showed that chloride ion reduces the corrosion resistance and the hydrocarbonate ions induce rapid surface passivation. Surface adsorption of amino acids increases polarization resistance and reduces current densities. Mg-Ca alloy showed higher corrosion rate in presence of proteins. Liu *et al.* [75] investigated the effect of albumin on *in vitro* degradation behavior of Mg-Ca1.5 alloy. Adsorption of albumin molecule on the surface led to decreased corrosion and hydrogen evolution rates. Filiform corrosion caused by chloride ions was also significantly inhibited due to surface adsorption of albumin. This inhibitive effect became stronger with higher concentrations of albumin in the solution.

## 8. Concluding Remarks

Magnesium-calcium (Mg-Ca) alloys have shown to be very promising in development of biodegradable, biocompatible, metallic orthopedic implants. Microstructure, mechanical properties, electrochemical behavior, and degradation kinetics of Mg-Ca implants are all affected by the amount of alloying element, *i.e.*, Ca. Beside alloying, surface coating and thermo-mechanical processing of the implants have been explored by researchers as potential ways to tackle high degradation kinetics of Mg-Ca implants in physiological environment and to postpone or to slow the degradation process. In this context, adjusting the kinetics of degradation through thermo-mechanical processing seems more advantageous. Surface mechanical treatment can tailor surface integrity of the orthopedic implant in such a way that not only their degradation rate matches the healing rate but also the mechanical properties and fatigue life of the implants improve. Although, there have been studies on Mg-Ca orthopedic products very recently, however, there is a lot left to be done in order to successfully realize Mg-Ca alloys as degradable orthopedic material in medical device manufacturing. Exact simulation of the physiology, surrounding a bone trauma in the human body, should be a major concern in future studies. There is still a considerable need to explore how surface integrity relates to bioperformance of Mg-Ca alloys. In this respect, isolating surface integrity characteristics (surface roughness, microhardness, residual stresses, and microstructure) and studying their effects on degradation kinetics of Mg-Ca implants remains as a major challenge for future studies. 

## References

[1] Bergen G., Chen L.H., Warner M., Fingerhut L.A. (2008). Injury in the United States: 2007 Chart Book.

[2] Feser K., Kietzmann M., Baumer W., Krause C., Bach F.W. (2011). Effects of degradable Mg-Ca alloys on dendritic cell function. J. Biomater. Appl..

[3] Li Z., Gu X., Lou S., Zheng Y. (2008). The development of binary Mg-Ca alloys for use as biodegradable materials within bone. Biomaterials.

[4] Thomann M., Krause C., Bormann D., von der Hoh N., Windhagen H., Meyer-Lindenberg A. (2009). Comparison of the resorbable magnesium alloys LAE442 und MgCa0.8 concerning their mechanical properties, their progress of degradation and the bone-implant contact after 12 months implantation duration in a rabbit model. Materialwiss. Werkst..

[5] Thomann M., Krause C., Angrisani N., Bormann D., Hassel T., Windhagen H., Meyer-Lindenberg A. (2010). Influence of a magnesium-fluoride coating of magnesium-based implants (MgCa0.8) on degradation in a rabbit model. J. Biomed. Mater. Rer. A.

[6] Von der Hoh N., Bormann D., Lucas A., Denkena B., Hackenbroich C., Meyer-Lindenberg A. (2009). Influence of different machining treatments of magnesium-based resorbable implants on the degradation behavior in rabbits. Adv. Eng. Mater..

[7] Krause A., von der Hoh N., Bormann D., Krause C., Bach F.W., Windhagen H., Meyer-Lindenberg A. (2010). Degradation behavior and mechanical properties of magnesium implants in rabbit tibiae. J. Mater. Sci..

[8] Amelfarzad H., Peivandi M.T., Yusofsani S.M.R. (2007). In body corrosion fatigue failure of a stainless steel orthopaedic implant with a rare collection of different damage mechanisms. Eng. Fail. Anal..

[9] Triantafyllidis G.K., Kazantzis A.V., Karageorgiou K.T. (2007). Premature fracture of a stainless steel 316L orthopedic plate implant by alternative episodes of fatigue and cleavage decoherence. Eng. Fail. Anal..

[10] Kanchanomai C., Phiphobmongkol V., Muanjan P. (2008). Fatigue failure of an orthopedic implant—A locking compression plate. Eng. Fail. Anal..

[11] Denkena B., Witte F., Podolsky C., Lucas A. Degradable implants made of magnesium alloys. Proceedings of the 5th Euspen International Conference.

[12] Denkena B., Lucas A. (2007). Biocompatible magnesium alloys as absorbable implant materials—Adjusted surface and subsurface properties by machining processes. Ann. CIRP.

[13] Benli S., Aksoy S., Havitcioglu H., Kucuk M. (2008). Evaluation of bone plate with low stiffness material in terms of stress distribution. J. Biomech..

[14] Completo A., Fonseca F., Simoes J.A. (2008). Strain shielding in proximal tibia of stemmed knee prosthesis: Experimental study. J. Biomech..

[15] Completo A., Fonseca F., Simoes J.A. (2008). Experimental evaluation of strain shielding in distal femur in revision TKA. Exp. Mech..

[16] Au A.G., Raso V.J., Liggins A.B., Amirfazli A. (2007). Contribution of loading conditions and material properties to stress shielding near the tibial component of total knee replacements. J. Biomech..

[17] Shi J.F., Wang C.J., Laoui T., Hart W., Hall R. (2007). A Dynamic model of simulating stress distribution in the distal femur after total knee replacement. Proc. Inst. Mech. Eng. Part H.

[18] Isaksson H., Lerner A.L. Mathematical modeling of stress shielding with bioresorbable materials for internal fracture fixation. Proceedings of the IEEE 29th Annual Bioengineering Conference.

[19] Nagels J., Stokdijk M., Rozing P.M. (2003). Stress shielding and bone resorption in shoulder arthroplasty. J. Should. Elb. Surg..

[20] Wolff J. (1986). The Law of Bone Remodeling.

[21] Gefen A. (2002). Computational simulations of stress shielding and bone resorption around existing and computer-designed orthopaedic screws. Med. Biol. Eng. Comput..

[22] Navarro M., Michiardi A., Castano O., Planell J.A. (2008). Biomaterials in orthopaedic. J. R. Soc. Interface.

[23] Hench L., Polak J. (2002). Third generation biomedical materials. Science.

[24] Seiler H.G. (1987). Handbook on Toxicity of Inorganic Compounds.

[25] Song G. (2007). Control of biodegradation of biocompatible magnesium alloys. Corros. Sci..

[26] Hassel T., Bach F.W., Kainer K.U. (2007). Production and properties of small tubes made from mgca0.8 for application as stent in biomedical science. Proceedings of the 7th International Conference on Magnesium Alloys and Their Applications.

[27] Hassel T., Bach F.W., Krause C., Kainer K.U. (2007). Influence of alloy composition on the mechanical and electrochemical properties of binary Mg-Ca alloys and its corrosion behavior in solutions at different chloride concentrations. Proceedings of the 7th International Conference on Magnesium Alloys and Their Applications.

[28] Kannan M.B., Raman R.K.S. (2008). *In vitro* degradation and mechanical integrity of calcium containing magnesium alloys in modified simulated body fluid. Biomaterials.

[29] Kirkland N.T., Birbilis N., Walker J., Woodfield T., Dias G.J., Staiger M.P. (2010). *In vitro* dissolution of magnesium-calcium binary alloys: Clarifying the unique role of calcium additions in bioresorbable magnesium implant alloys. J. Biomed. Mater. Res. B.

[30] Rad H.R.B., Idris M.H., Kadir M.R.A., Farahany S. (2012). Microstructure analysis and corrosion behavior of biodegradable Mg-Ca implant alloys. Mater. Des..

[31] Kainer K.U., Lach E., Mordike B.L., Kainer K.U. (1998). Deformation behavior of AZ alloys at high strain rates. Magnesium Alloys and Their Applications.

[32] Drynda A., Hassel T., Hoehn R., Perz A., Bach F.W., Peuster M. (2010). Development and biocompatibility of a novel corrodible fluoride-coated magnesium-calcium alloy with improved degradation kinetics and adequate mechanical properties for cardiovascular applications. J. Biomed. Mater. Res. A.

[33] El-Magd E., Abouridouane M., Kainer K.U. (2000). Compression test on magnesium alloy mgal8zn at high strain rates and temperatures. Magnesium Alloys and Their Applications.

[34] El-Magd E., Abouridouane M., Kainer K.U. (2004). Influence of strain rate and temperature on deformation and fracture behavior of magnesium alloy MgAl8Zn: tests and numerical simulations. Proceedings of the 6th International Conference on Magnesium Alloys and Their Applications.

[35] El-Magd E., Abouridouane M. (2006). Characterization, modeling and simulation of deformation and fracture behavior of the light-weight wrought alloys under high strain rate loading. Int. J. Impact Eng..

[36] Sealy M.P., Guo Y.B. (2010). Surface integrity and process mechanics of laser shock peening of novel biodegradable magnesium-calcium (Mg-Ca) alloy. J. Mech. Behav. Biomed..

[37] Guo Y.B., Salahshoor M. (2010). Process mechanics and surface integrity by high-speed dry milling of biodegradable magnesium-calcium implant alloys. CIRP Ann. Manuf. Tech..

[38] Salahshoor M., Guo Y.B. (2011). Cutting mechanics in high speed dry machining of biomedical magnesium-calcium alloy using internal state variable plasticity model. Int. J. Mach. Tools Manuf..

[39] El-Magd E., Abouridouane M. (2003). Influence of strain rate and temperature on the compressive ductility of Al, Mg, Ti alloys. J. Phys. Paris.

[40] El-Magd E., Scholles H., Weisshaupt H. (1996). Adiabatic flow curves of metallic materials at high strain rates. Mat. Wiss. U. Werkst..

[41] Ataya S., El-Magd E. (2007). Quasi-static behavior of Mg-alloys with and without short fiber reinforcement. Theor. Appl. Fract. Mech..

[42] Essa Y.E., Perez-Castellanos J.L. (2003). Effects of the strain rate and temperature on the mechanical behavior of a Mg-5%Zn alloy reinforced with SiC particles. J. Mater. Process. Tech..

[43] Gu X.N., Zheng W., Cheng Y., Zheng Y.F. (2009). A study on alkaline heat treated Mg-Ca alloy for the control of the biocorrosion rate. Acta Biomater..

[44] Zhang C.Y., Zeng R.C., Liu C.L., Gao J.C. (2010). Comparison of calcium phosphate coatings on Mg-Al and Mg-Ca alloys and their corrosion behavior in Hank’s solution. Surf. Coat. Tech..

[45] Li M., Chen Q., Zhang W., Hu W., Su Y. (2011). Corrosion behavior in SBF for titania coatings on Mg-Ca alloy. J. Mater. Sci..

[46] Wang H., Estrin Y., Zuberova Z. (2007). Bio corrosion of a magnesium alloy with different processing histories. Mater. Lett..

[47] Bach F.W., Denkena B., Weinert K., Alpers P., Bosse M., Hammer N. Influence of cutting and non-cutting processes on the corrosion behavior and the mechanical properties of magnesium alloys. Proceedings of the 7th International Conference on Magnesium Alloys and Their Applications.

[48] Bozdana A.T., Gindy N.N.Z. (2008). Comparative experimental study on effects of conventional and ultrasonic deep cold rolling processes on Ti-6Al-4V. Mater. Sci. Tech..

[49] Prevey P.S., Ravindranath R.A., Shepard M., Gabb T. (2006). Case studies of fatigue life improvement using low plasticity burnishing in gas turbine engine applications. J. Eng. Gas Turb. Power.

[50] Seemikeri C.Y., Brahmankar P.K., Mahagaonkar S.B. (2008). Low plasticity burnishing: An innovative manufacturing method for biomedical applications. J. Manuf. Sci. Eng..

[51] Prevey P., McNulty D., Carr J., Sade P., Craft A. Fatigue strength enhancement of Ti-6Al-4V ELI femoral distal stems using low plasticity burnishing. Proceedings of the ASM MPMD (Materials & Processes for Medical Devices) Conference and Expo.

[52] Disegi J.A., Sax C. Effect of low plasticity burnishing on the fatigue strength of spinal rods. Proceedings of the ASM MPMD (Materials & Processes for Medical Devices) Conference and Expo.

[53] Schuh A., Zeller C., Holzwarth U., Kachler W., Wilcke G., Zeiler G., Eigenmann B., Bigoney J. (2007). Deep rolling of titanium rods for application in modular total hip arthroplasty. J. Biomed. Mater. Res. B.

[54] Prevey P.S., Hornbach D.J., Jayaraman N. (2007). Controlled plasticity burnishing to improve the performance of friction stir processed Ni-Al bronze. Mater. Sci. Forum.

[55] Gill C.M., Fox N., Withers P.J. (2008). Shakedown of deep cold rolling residual stresses in titanium alloys. J. Phys. D.

[56] Tsuji N., Tanaka S., Takasugi T. (2008). Evaluation of surface-modified Ti-6Al-4V alloy by combination of plasma-carburizing and deep rolling. Mater. Sci. Eng. A.

[57] Tsuji N., Tanaka S., Takasugi T. (2009). Effect of combined plasma-carburizing and deep rolling on notch fatigue property of Ti-6Al-4V alloy. Mater. Sci. Eng. A.

[58] Nikitin I., Besel M. (2008). Correlation between residual stress and plastic strain amplitude during low cycle fatigue of mechanically surface treated austenitic stainless steel AISI 304 and ferritic-pearlitic steel SAE 1045. Mater. Sci. Eng. A.

[59] Nikitin I., Besel M. (2008). Residual stress relaxation of deep-rolled austenitic steel. Scr. Mater..

[60] Nikitin I., Altenberger I. (2007). Comparison of the fatigue behavior and residual stress stability of laser-shock peened and deep rolled austenitic stainless steel AISI 304 in the temperature range 25–600 °C. Mater. Sci. Eng. A.

[61] Juijerm P., Altenberger I. (2007). Effective boundary of deep rolling treatment and its correlation with residual stress stability of Al-Mg-Mn and Al-Mg-Si-Cu alloys. Scr. Mater..

[62] Jayaraman N., Hornbach D.J., Prevey P.S. Mitigation of fatigue and pre-cracking damage in aircraft structures through low plasticity burnishing. Proceedings of the USAF Aircraft Structural Integrity Program (ASIP).

[63] Scheel J.E., Hornbach D.J., Prevey P.S. Safe life conversion of aircraft aluminum structures via low plasticity burnishing for mitigation of corrosion related failures. Proceedings of the Preliminary Program for 2009 DoD Corrosion Conference.

[64] Lopez L.N., Lamikiz A., Sanchez J.A., Arana J.L. (2007). The effect of ball burnishing in heat treated steel and inconel 718 milled surfaces. Int. J. Adv. Manuf. Tech..

[65] Seemikeri C.Y., Brahmankar P.K., Mahagaonkar S.B. (2008). Investigations on surface integrity of AISI 1045 using LPB tool. Tribol. Int..

[66] Rao D.S., Hebbar H.S., Komaraiah M., Kempaiah U.N. (2008). Investigation on the effect of ball burnishing parameters on surface hardness and wear resistance of HSLA dual-phase steels. Mater. Manuf. Process..

[67] Pourbaix M. (1974). Atlas of Electrochemical Equilibria in Aqueous Solutions.

[68] Witte F., Kaese V., Haferkamp H., Switzer E., Meyer-Lindenberg A., Wirth C.J., Windhagen H. (2005). *In vivo* corrosion of four magnesium alloys and the associated bone response. Biomaterials.

[69] Staiger M.P., Pietak A.M., Huadmai J., Dias G. (2006). Magnesium and its alloys as orthopedic biomaterials: A review. Biomaterials.

[70] Miller D.L., Goswami T. (2007). A review of locking compression plate biomechanics and their advantages as internal fixators in fracture healing. Clin. Biomech..

[71] Tönshoff H.K., Friemuth T., Winkler J., Podolsky C. (2000). Improving the characteristics of magnesium workpieces by burnishing operations. Magnesium Alloys and Their Applications.

[72] Roseler B., Schiller C.A. (2001). Current-potential correlated noise measurement (CorrELNoise): A new technique for the evaluation of electrochemical noise analysis. Mater. Corros..

[73] Witte F., Fischer J., Maier P., Blawert C., Stormer M., Hort M., Kainer K.U. (2007). Magnesium-hydroxyapatite composites as an approach to degradable biomaterials. Proceedings of the 7th International Conference on Magnesium Alloys and Their Applications.

[74] Gu X.N., Zheng Y.F., Chen L.J. (2009). Influence of artificial biological fluid composition on the biocorrosion of potential orthopedic Mg-Ca, AZ31, AZ91 alloys. Biomed. Mater..

[75] Liu C.L., Wang Y.J., Zeng R.C., Zhang X.M., Huang W.J., Chu P.K. (2010). *In vitro* corrosion degradation behavior of Mg-Ca alloy in the presence of albumin. Corros. Sci..

